# Synthesis and Biological Evaluation of Novel Acenaphthene Derivatives as Potential Antitumor Agents

**DOI:** 10.3390/molecules16032519

**Published:** 2011-03-17

**Authors:** Yong-Mei Xie, Yi Deng, Xiao-Yun Dai, Jie Liu, Liang Ouyang, Yu-Quan Wei, Ying-Lan Zhao

**Affiliations:** State Key Laboratory of Biotherapy, West China Hospital, West China Medical School, Sichuan University, Chengdu, 610041, China; E-Mails: xieym@scu.edu.cn (Y.M.X.); xiaofeng2525@126.com (Y. D.); homcndc@163.com (X.Y.D); ljscsx2009@163.com (J. L.); klivis@163.com (L. O.)

**Keywords:** synthesis, acenaphthene derivatives, antitumour activity

## Abstract

Twelve novel acenaphthene derivatives have been synthesized. The structures of all compounds were confirmed by ^1^H-NMR, MS and elemental analysis. Their antitumor activities were evaluated in six human solid tumor cell lines, namely non-small cell lung cancer (H460), human colon adenocarcinoma (SW480), human breast cancer cell (MDA-MB-468 and SKRB-3), human melanoma cell (A375) and human pancreatic cancer (BxPC-3). Among them, compound **3c** shows the best antitumor activity against SKRB-3 cell line, as high as the positive control adriamycin.

## 1. Introduction

Acenaphthene derivatives have gained great importance due to their diverse biological properties including antitumor [1,2], antifungal [3], antimicrobial [4], anti-inflammatory [5,6] and insecticidal [7] activities. In order to obtain novel acenaphthene derivatives with a wide spectrum of pharmaceutical applications, we report herein the synthesis of a series of acenaphthene derivatives containing thiazole backbone and the results of their preliminary *in vitro* antitumor evaluation.

## 2. Results and Discussion

### 2.1. Chemistry

**Scheme 1:**
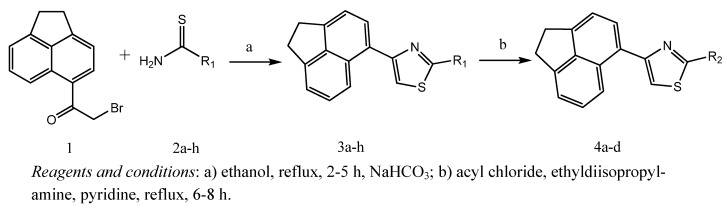
Synthetic route for **3a-h** and **4a-d**.

The synthetic route for the target compounds is shown in Scheme 1. The prepared acenaphthene derivatives **3a-h** and **4a-d** are listed in Table 1.

Commercially available acenaphthene was first reacted with bromoacetyl bromide in the presence of aluminum chloride to give crude 5-bromoacetylacenaphthene (**1**) (containing a little 3-bromoacetyl acenaphthene). The raw product was purified using a silica column with ethyl acetate/petroleum ether (1:15 v/v) as eluent. 3-Bromoacetylacenaphthene was eluted first and 5-bromoacetylacenaphthene was elueted next. The compounds **3a-h** were prepared by reacting **1** and 2-thiourea (**2a**) or aryl thioureas **2b-h** under reflux using ethanol as solvent. The HBr produced was neutralized with NaHCO_3_ solution and the crude product was recrystallized from appropriate solvent to yield **3a-h** (purity >97%, HPLC). Compound **3a** was further reacted with an appropriate acyl chloride in the presence of ethyldiisopropylamine using pyridine as solvent. Compounds **4a-b** (purity >97%, HPLC) were obtained by recrystallization of the crude products from appropriate solvents without purification by column chromatography.

### 2.2. Antiproliferative Activities

In the present study, the antiproliferative activity of the synthesized compounds was tested *in vitro* on six human tumor cell lines, including non-small cell lung cancer (H460), human colon adenocarcinoma (SW480), human breast cancer cell (MDA-MB-468 and SKRB-3), human melanoma cell (A375) and human pancreatic cancer (BxPC-3) by the MTT assay with adriamycin (ADM) as a positive control. The inhibition rates under drug concentration of 20 μM are summarized in Table 1. Among the acenaphthene derivatives, **3c** shows better inhibition against breast cancer cells line of MDA-MB-468 and SKRB-3 (inhibition rate are 55.5 ± 3.8% and 66.1 ± 2.2% respectively), while the corresponding results for ADM are 63.4 ± 0.4% and 68.1 ± 1.3%. These results suggest that **3c** has potent antitumor activity.

## 3. Experimental 

### 3.1. General

The human cancer cell lines were purchased from the American Type Culture Collection (ATCC, Rockville, MD, USA). Dulbecco’s modified Eagle medium (DMEM) and RPMI 1640 were purchased from Gibco (Grand Island, New York, USA). Fetal bovine serum (FBS) was purchased from Hyclone (Logan, Utah, USA). All chemicals were commercially available and used without further purification unless otherwise stated. Column chromatography was carried out on silica gel (200-300 mesh, Qingdao Marine Chemical Ltd., Qingdao, China). Thin layer chromatography (TLC) was performed on TLC silica gel 60 F254 plates. The purity of compound screened in biological assays was determined to be ≥97% by HPLC analysis with a photodiode array detector (Waters, Milford, MA, USA). An atlantis C_18_ (150 mm × 4.6 mm, i.d. 5 μm) (Waters, Milford, Mass, USA) was used with a gradient elution of methanol and HPLC-grade water as mobile phase at a flow rate of 1 mL/min. ^1^H-NMR were recorded at 400 MHz on a Varian spectrometer (Varian, Palo Alto, CA, USA) model Gemini 400 and chemical shifts (δ) are reported in parts per million relative to tetramethylsilane (TMS) used as an internal standard, where (δ) TMS = 0.00 ppm. Mass Spectra (MS) were measured by Q-TOF Priemier mass spectrometer utilizing electrospray ionization (ESI) (Micromass, Manchester, UK). Elemental analyses were carried out on a Carlo Erba-1106 analyzer. Melting points were determined on a SGW X-4 microscopic melting point (Shanghai Precision & Scientific Instrument Co., Ltd, China).

### 3.2. Preparation of 5-Bromoacetylacenaphthene *(**1**)*

5-Bromoacetylacenaphthene was synthesized according to a literature method [8] with some modifications. Briefly, aluminum chloride (22.8 g, 171 mmol) was added slowly while stirring to a solution of acenaphthene (18.6 g, 211 mmol) and bromoacetyl bromide (12.6 mL, 145 mmol) in CH_2_Cl_2_ (120 mL) at -20 °C. The mixture was allowed to warm to room temperature and stirred for 30 min, neutralized with 5% NaHCO_3_ solution, extracted with chloroform (3×50 mL), washed with water (2×50 mL) and dried over Na_2_SO_4_. The solvent was evaporated and residue was purified with column chromatography (ethyl acetate: petroleum ether = 1:15) to give compound **1** as an off-white solid. Yield: 13.8 g, 41.5%. ^1^H-NMR (CDCl_3_) δ: 3.43 (s, 4H), 4.58 (s, 2H), 7.33 (d, 1H, *J* = 7.6 Hz), 7.40 (d, 1H, *J* = 6.8 Hz), 7.64 (dd, 1H, *J*_1_ = 7.2 Hz, *J*_2_ = 7.2 Hz), 8.09 (d, 1H, *J* = 7.6 Hz), 8.69 (d, 1H, *J* = 8.4 Hz).

### 3.3. General Procedure for Preparing Compounds ***3a-h***

A mixture of compound **1** (0.50 mmol), **2a-h** (0.55 mmol; **2b-h** were prepared according to a literature method [9]) was refluxed in ethanol (20 mL). After completion of the reaction, the reaction mixture was evaporated. 5% NaHCO_3_ solution (5 mL) was added to the residue and the mixture was stirred for 20 min. The mixture was filtered and the obtained solid was recrystallized from solvent to give **3a-h**.

*4-(1,2-Dihydroacenaphthylen-5-yl)-1,3-thiazol-2-amine* **(3a**). Off-white solid (from ethanol); Yield: 55 mg, 43.8%; HPLC: 98.2%; m.p. 155.8-157.3 °C; ^1^H-NMR (DMSO) δ: 3.46 (m, 4H), 7.04 (s, 1H), 7.42 (dd, 2H, *J*_1_ = 4.0 Hz, *J*_2_ = 3.6 Hz), 7.56-7.81 (m, 3H), 8.92 (s, 2H); LC-MS: 253.4 (M+H)^+^; Anal. Calcd. for C_15_H_12_N_2_S (252.07): C, 71.40; H, 4.79; N, 11.10%. Found: C, 71.38; H, 4.81; N, 11.13%.

*N-Phenyl-4-(1,2-dihydroacenaphthylen-5-yl)-1,3-thiazol-2-amine* (**3b**). White solid (from ethanol/ water = 1:1); Yield: 97 mg, 59.1%; HPLC: 98.8%; m.p. 129.5-130.9 °C; ^1^H-NMR (CDCl_3_) δ: 3.43 (d, 4H, *J* = 2.0 Hz), 6.80 (s, 1H), 7.06-7.09 (m, 1H), 7.31-7.38 (m, 5H), 7.49 (m, 1H), 7.77 (d, 2H, *J* = 7.2 Hz), 8.18 (d, 1H, *J* = 8.4 Hz); LC-MS: 329.6 (M+H)^+^; Anal. Calcd. for C_21_H_16_N_2_S (328.43): C, 76.80; H, 4.91; N, 8.53%. Found: C, 76.83; H, 4.90; N, 8.52%.

*N-(4-Ethoxyphenyl)-4-(1,2-dihydroacenaphthylen-5-yl)-1,3-thiazol-2-amine* (**3c**). Gray solid (from ethanol); Yield:120 mg, 64.4%; HPLC: 99.3%; m.p. 194.3-197.0 °C; ^1^H-NMR (CDCl_3_) δ: 1.43 (t, 3H, *J* = 6.8 Hz), 3.42(dd, 4H, *J*_1_ = 3.2 Hz, *J*_2_ = 3.2 Hz), 4.03 (dd, 2H, *J*_1_ = 6.8 Hz, *J*_2_ = 7.2 Hz), 6.70 (s, 1H), 6.86 (d, 2H, *J* = 8.8 Hz), 7.24-7.31 (m, 4H), 7.48 (t, 1H), 7.57 (sbr, 1H), 7.74 (d, 1H, *J* = 7.2 Hz), 8.17 (d, 1H, *J* = 8.4 Hz); LC-MS: 373.4 (M+H)^+^; Anal. Calcd. for C_23_H_20_N_2_OS (372.48): C, 74.16; H, 5.41; N, 7.52%. Found: C, 74.15; H, 5.44; N, 7.51%.

*N-(4-Methoxyphenyl)-4-(1,2-dihydroacenaphthylen-5-yl)-1,3-thiazol-2-amine* (**3d**). Gray solid (from ethanol); Yield: 94 mg, 52.4%; m.p. 185.4-188.6 °C; HPLC: 99.6%; ^1^H-NMR (DMSO) δ: 3.41(s, 4H), 3.73 (s, 3H), 6.94 (d, 2H, *J* = 8.8 Hz), 7.09 (s, 1H), 7.36-7.62 (m, 5H), 7.79 (d, 1H, *J* = 7.2, Hz), 8.22 (d, 1H, *J* = 7.2 Hz), 10.21 (s, 1H); LC-MS: 359.5 (M+H)^+^; Anal. Calcd. for C_22_H_18_N_2_OS (358.46): C, 73.71; H, 5.06; N, 7.82%. Found: C, 73.75; H, 5.07; N, 7.80%. 

*N-(3,5-Dichlorophenyl)-4-(1,2-dihydroacenaphthylen-5-yl)-1,3-thiazol-2-amine* (**3e**). White solid (from ethanol); Yield: 109 mg, 54.9%; HPLC: 98.6%; m.p. 222.6-225.1 °C; ^1^H-NMR (DMSO) δ: 3.40 (s, 4H), 7.13 (s, 1H), 7.32 (s, 1H), 7.37-!7.54 (m, 3H), 7.81 (d, 1H, *J* = 7.2 Hz), 7.85 (d, 2H, *J* = 1.6 Hz), 8.31 (d, 1H, *J* = 8.4 Hz), 10.78 (s, 1H); LC-MS: 397.3 (M+H)^+^; Anal. Calcd. for C_21_H_14_N_2_S (397.32): C, 63.48; H, 3.55; N, 7.05%. Found: C, 63.45; H, 3.57; N, 7.06%.

*N-(4-Chlorophenyl)-4-(1,2-dihydroacenaphthylen-5-yl)-1,3-thiazol-2-amine* (**3f**). White solid (from ethanol); Yield: 105 mg, 57.9%; HPLC: 99.5%; m.p. 211.4-214.5 °C; ^1^H-NMR (CDCl_3_) δ: 3.41 (s, 4H), 6.78 (s, 1H), 7.15-7.30 (m, 5H), 7.47 (dd, 1H, *J*_1_ = 7.2 Hz, *J*_2_ = 7.2 Hz), 7.72 (d, 2H, *J* = 7.2 Hz), 8.08 (sbr, 1H), 8.13 (d, 1H, *J* = 8.4 Hz); LC-MS: 363.5 (M+H)^+^; Anal. Calcd. for C_21_H_15_ClN_2_S (362.88): C, 69.51; H, 4.17; N, 7.72%. Found: C, 69.50; H, 4.19; N, 7.71%. 

*4-(4-(1,2-Dihydroacenaphthylen-5-yl)-1,3-thiazol-2-ylamino) benzoic acid* (**3g**). White solid (from ethanol); Yield: 137 mg, 73.6%; HPLC: 99.0%; m.p.323.1-325.6 °C; ^1^H-NMR (DMSO) δ: 3.40 (s, 4H), 7.28 (s, 1H), 7.38 (m, 2H), 7.53 (dd, 1H, *J*_1_ = 7.2 Hz, *J*_2_ = 7.2 Hz), 7.80-7.91 (m, 5H), 8.24 (d, 1H, *J* = 8.4 Hz), 10.74 (s, 1H), 12.52 (s, 1H); LC-MS: 373.4 (M+H)^+^; Anal. Calcd. for C_22_H_16_N_2_O_2_S (372.44): C, 70.95; H, 4.33; N, 7.52%. Found: C, 70.91; H, 4.35; N, 7.54%.

*Methyl 2-(4-(1,2-dihydroacenaphthylen-5-yl)thiazol-2-ylamino)benzoate* (**3h**). White solid (from ethanol); Yield: 122 mg, 63.1%; HPLC: 99.1%; m.p. 258.4-260.7 °C; ^1^H-NMR (DMSO) δ: 3.43 (s, 4H), 4.93 (s, 3H), 7.15 (d, 2H, *J* = 8.0 Hz), 7.35-7.66 (m, 5H), 8.00 (d, 1H, *J* = 8.0 Hz), 8.35 (d, 1H, *J* = 8.4 Hz), 8.46 (d, 1H, *J* = 7.2 Hz), 12.72 (s, 1H); LC-MS: 387.6 (M+H)^+^; Anal. Calcd. for C_23_H_18_N_2_O_2_S (386.47): C, 71.48; H, 4.69; N, 7.25%. Found: C, 71.45; H, 4.70; N, 7.28%.

### 3.4. General Procedure for Preparing Compounds ***4a-d***

A mixture of **3a** (0.50 mmol), acyl chloride (0.55 mmol) and ethyldiisopropylamine (0.3 mmol) was refluxed in pyridine (10 mL). After completion of the reaction, the reaction mixture was evaporated. Water (20 mL) and ethyl acetate (50 mL) were added. The organic layer was washed with 5% Na_2_CO_3_ solution (20 mL), water and saturated sodium chloride solution, then dried over Na_2_SO_4_. The filtrate was evaporated and the obtained solid was recrystallized from solvent to give **4a-d**. 

*N-(4-(1,2-dihydroacenaphthylen-5-yl)-1,3-thiazol-2-yl) acetamide* (**4a**). White solid (from ethanol/ ethyl acetate = 2:1); Yield: 86 mg, 58.4%; HPLC: 98.1%; m.p. 192.5-193.8 °C; ^1^H-NMR (CDCl_3_) δ: 1.73 (s, 3H), 3.44 (s, 4H), 7.13 (s, 1H), 7.33 (dd, 2H, *J*_1_ = 4.0 Hz, *J*_2_ = 4.0 Hz), 7.47 (q, 1H, *J*_1_ = 6.8 Hz, *J*_2_ = 6.8 Hz), 7.72 (d, 1H, *J* = 7.2 Hz), 8.09 (d, 1H, *J* = 8.4 Hz), 10.70 (s, 1H); LC-MS: 295.4 (M+H)^+^; Anal. Calcd. for C_17_H_14_N_2_OS (294.37): C, 69.36; H, 4.79; N, 9.52%. Found: C, 69.40; H, 4.78; N, 9.50%.

*N-(4-(1,2-dihydroacenaphthylen-5-yl)-1,3-thiazol-2-yl)benzamide* (**4b**). Off-white solid (from ethanol/ water = 4:1); Yield: 79 mg, 44.3%; HPLC: 98.6%; m.p. 173.2-176.1 °C; ^1^H-NMR (CDCl_3_) δ: 3.37 (dd, 4H, *J*_1_ = 7.6 Hz, *J*_2_ = 8.0 Hz), 7.19-7.43 (m, 5H), 7.61 (d, 1H, *J* = 7.2 Hz), 7.72 (d, 2H, *J* = 7.6 Hz), 8.01 (d, 1H, *J* = 8.4 Hz), 11.06 (s, 1H); LC-MS: 357.4 (M+H)^+^; Anal. Calcd. for C_22_H_16_N_2_OS (356.44): C, 74.13; H, 4.52; N, 7.86%. Found: C, 74.15; H, 4.50; N, 7.88%. 

*N-(4-(1,2-dihydroacenaphthylen-5-yl)-1,3-thiazol-2-yl)-4-methoxybenzamide* (**4c**). White solid (from ethanol/ ethyl acetate = 2:1); Yield: 130 mg, 67.3%; HPLC: 99.5%; m.p. 210.6-213.8 °C; ^1^H-NMR (CDCl_3_) δ: 3.35 (dd, 4H, *J*_1_ = 7.6 Hz, *J*_2_ = 7.2 Hz), 3.73 (s, 3H), 6.62 (d, 2H, *J* = 8.8 Hz), 7.17-7.28 (m, 3H), 7.40 (m, 1H), 7.61 (t, 3H, *J* = 7.2 Hz), 7.99 (d, 1H, *J* = 8.4 Hz), 11.37 (s, 1H); LC-MS: 387.5 (M+H)^+^; Anal. Calcd. for C_23_H_18_N_2_O_2_S (386.47): C, 71.48; H, 4.69; N, 7.25%. Found: C, 71.47; H, 4.70; N, 7.23%.

*N-(4-(1,2-dihydroacenaphthylen-5-yl)-1,3-thiazol-2-yl)furan-2-carboxamide* (**4d**). White solid (from ethanol/ ethyl acetate = 2:1); Yield: 93 mg, 53.7%; HPLC: 98.7%; m.p. 184.9-187.4 °C; ^1^H-NMR (CDCl_3_) δ: 3.43 (d, 4H, *J* = 2.8 Hz), 5.58 (dd, 1H, *J*_1_ = 1.6 Hz, *J*_2_ = 1.6 Hz), 7.19 (s, 1H), 7.32-7.53 (m, 5H), 7.73 (d, 1H, *J* = 7.2 Hz), 8.11 (d, 1H, *J* = 8.4 Hz), 9.98 (s, 1H); LC-MS: 347.6 (M+H)^+^; Anal. Calcd. for C_20_H_14_N_2_O_2_S (346.40): C, 69.35; H, 4.07; N,8.09%. Found: C, 69.32; H, 4.05; N, 8.11%.

### 3.5. Cell Culture

Cell lines MDA-MB-468, SKRB-3 and A375 were maintained in Dulbecco’s modified Eagle medium (DMEM) containing 10% fetal bovine serum (FBS), penicillin (100 U/mL) and streptomycin (10 mg/L). Cell lines H460, SW480, and BxPC-3 were maintained in RPMI 1640 containing 10% FBS, penicillin (100 U/mL) and streptomycin (10 mg/L). Cells were grown in a 5% CO_2_ incubator at 37 °C.

### 3.6. Cell Proliferation Assay (MTT Assay)

Cells (3-5 × 10^3^/well) were seeded in 200 µL of medium/well in 96-well plates (Costar Corning, Rochester, NY). After incubation overnight, the compounds dissolved in dimethylsulfoxide (DMSO) were added to final concentration of 20 μM, adriamycin (ADM) used as a positive control. Cells were exposed to the compounds for 48 h. After incubation with 3-(4,5-dimethylthiazol-2-yl)-2,5-diphenyltetrazolium bromide (MTT; 0.5 mg/mL) for 4 h, the medium was removed and 150 μL of DMSO was added to dissolve formazan crystals. Absorbance was measured at 570 nm using an ELISA reader (Thermo). The effects of the compounds on the proliferation of cancer cells were expressed as the % cell growth inhibition, using the following formula: % inhibition = (A_570_ of control - A_570_ of treated cells)/A_570_ of control cells × 100%.

## 4. Conclusions

In this work, twelve novel acenaphthene derivatives containing thiazole backbone were synthesized and the *in vitro* antitumor activity was evaluated. Among all of these derivatives, compound **3c** showed more potent cytotoxicity towards SKRB-3 cell than the positive control adriamycin, which indicates the potential of **3c** as a future anticancer agent.

## Figures and Tables

**Table 1:** The substituents and antitumor activities of **3a-h** and **4a-d**.*

Compd.	R_1_	R_2_	Inhibition rate (%)					
			H460	SW480	MDA-MB-468	SKRB-3	A375	BxPC-3
**3a**	-NH_2_	-	25.2±2.8	1.7±2.3	0.8±3.9	1.3±8.8	42.3±2.2	4.0±3.4
**3b**	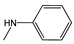	-	19.3±6.6	20.2±5.6	27.3±6.8	35.2±3.2	25.7±3.2	18.6±2.8
**3c**	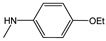	-	24.3±9.1	22.6±3.0	55.5±3.8	66.1±2.2	31.7±5.0	17.1±3.7
**3d**	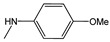	-	12.2±4.0	11.1±2.5	31.6±8.1	42.4±2.9	18.2±9.2	6.1±5.5
**3e**	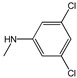	-	20.6±5.2	0.4±2.6	15.2±7.3	20.7±4.4	0.5±1.5	2.6±6.6
**3f**	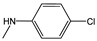	-	33.8±1.6	30.7±0.5	25.9±1.5	47.5±2.8	10.5±1.0	20.6±2.4
**3g**	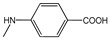	-	26.2±2.1	1.9±2.6	5.2±3.7	34.7±6.6	4.9±3.1	1.1±7.8
**3h**	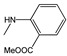	-	43.7±1.3	41.1±2.7	30.6±0.6	41.6±2.4	9.1±1.6	32.1±1.0
**4a**	-	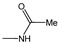	12.1±0.5	14.6±1.4	20.5±1.6	31.0±4.8	22.6±2.5	10.2±1.7
**4b**	-	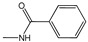	17.6±0.8	33.8±4.0	31.3±1.3	41.7±5.2	35.0±6.6	20.2±1.4
**4c**	-	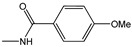	19.3±3.2	16.3±0.4	13.2±5.0	26.3±3.0	16.6±7.1	11.4±3.6
**4d**	-	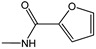	35.1±3.3	22.7±1.0	25.3±1.9	38.0±2.8	39.7±1.9	25.0±3.5
**ADM**	-	-	63.3±0.9	46.1±0.4	63.4±0.4	68.1±1.3	70.4±2.0	39.4±0.7

* Results are given in concentrations of 20 μM after a continuous exposure of 48 h and show means±SEM of three-independent experiments.
